# A Light Multi-View Stereo Method with Patch-Uncertainty Awareness

**DOI:** 10.3390/s24041293

**Published:** 2024-02-17

**Authors:** Zhen Liu, Guangzheng Wu, Tao Xie, Shilong Li, Chao Wu, Zhiming Zhang, Jiali Zhou

**Affiliations:** 1 College of Science, Zhejiang University of Technology, Hangzhou 310023, China; zhenliu@zjut.edu.cn (Z.L.); 2112109013@zjut.edu.cn (G.W.); 211122090015@zjut.edu.cn (T.X.); 211122090017@zjut.edu.cn (S.L.); wuchao@zjut.edu.cn (C.W.); 2 Rept Battero, Wenzhou 325058, China; zhzhm2@163.com

**Keywords:** multi-view stereo, attention mechanism, cost volume, depth learning

## Abstract

Multi-view stereo methods utilize image sequences from different views to generate a 3D point cloud model of the scene. However, existing approaches often overlook coarse-stage features, impacting the final reconstruction accuracy. Moreover, using a fixed range for all the pixels during inverse depth sampling can adversely affect depth estimation. To address these challenges, we present a novel learning-based multi-view stereo method incorporating attention mechanisms and an adaptive depth sampling strategy. Firstly, we propose a lightweight, coarse-feature-enhanced feature pyramid network in the feature extraction stage, augmented by a coarse-feature-enhanced module. This module integrates features with channel and spatial attention, enriching the contextual features that are crucial for the initial depth estimation. Secondly, we introduce a novel patch-uncertainty-based depth sampling strategy for depth refinement, dynamically configuring depth sampling ranges within the GRU-based optimization process. Furthermore, we incorporate an edge detection operator to extract edge features from the reference image’s feature map. These edge features are additionally integrated into the iterative cost volume construction, enhancing the reconstruction accuracy. Lastly, our method is rigorously evaluated on the DTU and Tanks and Temples benchmark datasets, revealing its low GPU memory consumption and competitive reconstruction quality compared to other learning-based MVS methods.

## 1. Introduction

Multi-view stereo (MVS), as a 3D reconstruction method, plays a crucial role in 3D computer vision, with various applications such as in virtual reality, augmented reality, and autonomous driving. Taking a sequence of images from different viewpoints and the corresponding camera parameters as the input, multi-view stereo can estimate each pixel’s depth information and generate the corresponding 3D representation of the observed scene. As a pivotal issue in 3D computer vision, multi-view stereo has garnered extensive research attention [1,2,3,4].

With the rapid development of deep learning technologies in computer vision, learning-based multi-view stereo methods have produced advanced results [4,5,6] in recent years. Learning-based multi-view stereo algorithms generally consist of several components, including feature extraction, depth sampling, cost volume construction, cost volume regularization, and depth regression. However, significant GPU memory requirements not only limit image processing to low resolutions but also hamper the adoption of multi-view stereo on various edge computing devices. In practical applications of 3D vision, the devices deployed often possess limited computational resources. For instance, in autonomous driving scenarios, Lidar data are typically processed using three-dimensional point cloud compression techniques to mitigate storage and transmission costs [7]. Unlike Lidar data processing, the primary computational challenge for multi-view stereo lies in generating point clouds from two-dimensional images and camera parameters given an input source. Therefore, reducing the algorithm’s memory consumption could substantially enhance the practicality of this technology. Recently, many researchers have proposed improved approaches to deal with the problem of the high computation of learning-based multi-view stereo methods. In particular, a coarse-to-fine architecture has been widely used to design efficient multi-view stereo networks [6,8,9,10,11,12]. Generally, in these methods, an initial cost volume is usually constructed at a low resolution rather than at a fixed resolution, then a new cost volume is built at a higher resolution iteratively with the last stage result and finally, a depth map is obtained. Progressively narrowing the hypothesis of the depth plane in different stages [6,8,9,10,11,12] is also a key strategy to reduce the amount of computation. Despite the significance of the coarse-stage outputs as an input in the fine-stage cost volume construction, having an influence on the final results, these existing approaches need to pay more attention to the feature information at the coarse stage. If the feature extraction phase in the coarse stage is inadequate, the poor initial result may adversely impact the final results in subsequent stages and the final outputs. However, an intensive feature extraction step always increases the computational load and GPU consumption, and it remains a challenge to balance accuracy and computational efficiency.

Furthermore, another existing challenge in cascade-based multi-view stereo is adapting the depth hypothesis range. In the initial stage, plane sweeping covers the entire conceivable depth range. Simultaneously, during depth hypothesis generation for finer stages in many cascade-based algorithms [6,8,10,12], the estimated depth values from the previous stage are used as the sampling interval’s center, with a fixed sampling distance for each pixel within its respective stage. Nevertheless, setting a uniform sampling distance for each pixel is not an ideal approach because the optimization in the depth refinement stage varies across different pixels in the same depth map, where some pixels may have stabilized depths and others may exhibit significant variations. Considering this challenge, Cheng [11] utilized the probability distribution at each pixel to set the sampling distance; however, this approach demonstrates a poor performance in GPU memory usage and running time, while its training time is also huge.

**Figure 1:**
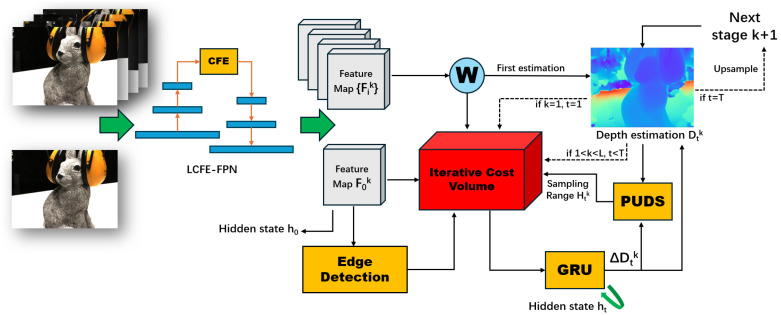
Overview of our method. In this figure, we take the *t*-th optimization at the *k*-th resolution stage as an example. We first utilize LCFE-FPN to extract multi-scale features from input images and enhance features at the initial stage. Taking the last depth estimation $D_{t}^{k-1}$ as the input, we construct an iterative cost volume and employ GRUs to optimize it. After the *t*-th optimization at *k*-th stage, the depth offset $\Delta D_{t}^{k}$ for refinement is calculated, and the next sampling range $H_{t}^{k}$ is also configured adaptively. If the optimization time *t* equals the pre-configured *T*, the updated depth estimation $D_{t}^{k}$ will be upsampled to $D_{1}^{k+1}$ for GRU optimization in the next (k+1-th) stage.

In this paper, we present a lightweight multi-view stereo method that incorporates a patch-uncertainty-based depth sampling strategy. The overall framework of our proposed method is shown in Figure 1. In the feature extraction step, we introduce a light coarse-feature-enhanced feature pyramid network (LCFE-FPN). This network mitigates GPU memory consumption and improves the final reconstruction accuracy. The LCFE-FPN is structured as a feature pyramid network [13], with a novel coarse-feature-enhanced module (CFE) integrating both channel and spatial attention mechanisms designed for the lowest-resolution stage. We also replace the original batch normalization layers with Inplace-ABN [14] layers, thereby further reducing GPU memory usage in the classical FPN. In the subsequent depth refinement step, we propose a patch-uncertainty-based depth sampling strategy (PUDS). The PUDS utilizes the patch depth variations in the estimated depth map during depth refinement to adaptively modify the inverse depth sampling distance for each pixel at the patch level. With each iteration of the GRU-based optimization, this inverse depth sampling strategy allocates a broader depth sampling range for regions with significant depth variations and a narrower range for regions with minor variations. Furthermore, during the GRU-based optimization, we construct an edge-aware iterative cost volume that contains four different types of features: content features, edge features, geometric features, and depth features. The incorporation of an edge detection operator [15] to extract supplementary edge information and integrate it into the iterative cost volume [16] further improves the quality of the final 3D point cloud. To evaluate our introduced method, we choose two challenging benchmark datasets: the DTU dataset [17] and the Tanks and Temples dataset [18]. Our proposed method demonstrates a reduced GPU memory usage and competitive reconstruction quality when compared to various other learning-based MVS methods [1,4,5,6,8,9,10,11,19,20,21,22,23,24].

In summary, our main contributions are as follows:

- We design a network (LCFE-FPN) based on a novel coarse-feature-enhanced module (CFE) for the feature extraction step, which not only improves the accuracy of the final reconstruction result but also reduces the GPU memory consumption.
- We propose a patch-uncertainty-based depth sampling strategy (PUDS) for the depth refinement step. This sampling strategy calculates patch-wise variation based on each pixel’s depth variation and assigns different sampling ranges adaptively for each pixel. Specifically, we aggregate edge information extracted by an edge-detection operator into the iterative cost volume construction, enhancing the model’s performance.
- We perform extensive experiments on two challenging datasets (the DTU dataset [17] and the Tanks and Temples dataset [18]), our approach achieves a competitive performance in both GPU consumption and reconstruction quality.

## 2. Related Work

### 2.1. Traditional Multi-View Stereo Methods

Multi-view stereo (MVS), as a fundamental problem in the field of 3D reconstruction in computer vision, addresses the spatial geometry recovery of scenes from photographs. It had garnered significant attention and made substantial progress before the advent of deep learning. Traditional multi-view stereo methods can be broadly categorized into the following four types: voxel-based method [25,26,27,28,29], mesh-based methods [30,31], surfel-based methods [19,32,33] and depth-map-based methods [1,20,21,34,35]. Among these four methods, the voxel-based method partitions the space into a group of voxels, requiring extremely high memory consumption. The mesh-based method is less robust, as its final reconstruction performance relies on its initialization. Meanwhile, the surfel-based method represents surfaces as a set of surfels, and is simple but efficient. However, the surfel-based method entails additional cumbersome post-processing steps to generate the final 3D model. Depth-map-based methods compute depth values for each pixel from each image, reproject the pixel into the 3D space, and fuse these points to generate a point cloud model. Of these four methods, the depth-map-based approach is the most flexible and is most extensively applied in the field. Over recent years, depth-map-based methods have achieved significant success, and there are great algorithmic frameworks in use, such as Furu [19], Gipuma [21], Tola [20], and COLMAP [1]. Despite the commendable performance of traditional multi-view stereo, the following drawbacks still needs to be improved: high computational requirements, slow processing speeds, and suboptimal handling of scenarios with weak textures or highly reflective patches.

### 2.2. Learning-Based Multi-View Stereo Methods

Recently, with the integration of deep learning, the learning-based multi-view stereo method has experienced rapid development and achieved outstanding performance. Yao [4] introduced MVSNet, the first end-to-end learning-based multi-view stereo network, laying the foundation for fast growth in the coming years. MVSNet [4] employs a shared-weight 2D-CNN network to extract feature maps from input images. The differential homography transformation [36] is then applied to warp these feature maps to the reference perspective. This method utilizes a series of depth hypothesis planes to construct a cost volume, representing the correlation between the source and reference images. Subsequently, a 3D-CNN network is employed for cost volume regularization. In the end, the output is obtained as the estimated depth map of the reference image through depth regression. In DTU benchmark datasets [17], MVSNet [4] not only outperforms previous traditional MVS methods [1,19,20], but also has a much faster runtime. However, due to the high GPU memory consumption, only low-resolution images can be used as the input images in MVSNet. Many learning-based MVS methods have been proposed to deal with the problem of GPU memory consumption. Yao [22] proposed the improved approach R-MVSNet [22], which replaces the 3D-CNN network in depth refinement with a sequence of GRU convolutions. This improvement reduces the GPU memory consumption and makes it feasible for 3D reconstruction at a high resolution. Gu [6] proposed the CasMVSNet model, which constructs cascade cost volumes based on a feature pyramid network (FPN) [13]. Benefiting from its novel coarse-to-fine architecture, CasMVSNet can deal with the input images from the DTU dataset [17] at raw resolutions. Similar to CasMVSNet [6], CVP-MVSNet [8] and Fast-MVS [23] also contain a coarse-to-fine framework, and both have demonstrated a great performance on benchmark datasets [17,18]. Based on the coarse-to-fine cascade framework, UCS-Net [11] further introduces a depth sampling strategy that utilizes uncertainty estimations to adaptively produce spatially varying depth hypotheses. Uncertainty is also used by Vis-MVSNet [9] to explicitly infer and integrate the pixel-wise occlusion information in multi-view cost volume fusion. PatchMatch [2], as a classical and traditional stereo-matching algorithm, was also integrated into the learning-based MVS framework, and the resulting model was named PatchmatchNet [2]. Recently, Effi-MVS [10] was proposed, demonstrating a novel way to construct dynamical cost volumes in depth refinement. In addition, TransMVSNet [37] is the first learning-based MVS method that leverages the Transformer [38] to realize robust, long-range global context aggregation within and across images. Compared with these existing learning-based MVS methods [4,5,6,8,9,10,11,22,23,24], our proposed method not only demonstrates a lower GPU memory consumption and a faster running time, but also delivers a competitive performance in terms of the reconstruction quality.

## 3. Methodology

In the context of our learning-based multi-view stereo (MVS) framework, we leverage the reference image $I_{0}$ and its adjacent source images ${\left\{I_{i}\right\}}_{i=1,2\ldots N-1}$, along with their corresponding camera intrinsic and extrinsic parameters. Our method enables the rapid prediction of an accurate depth map for $I_{0}$. As illustrated in Figure 1, our method consists of a three steps: multi-scale feature extraction, depth estimation, and depth refinement. During the feature extraction step, we introduce a light coarse-feature-enhanced feature pyramid network (LCFE-FPN), designed to reduce GPU memory consumption and enhance the feature map at the coarse stage, thereby improving the overall performance. In the subsequent depth estimation step, we propose a patch-uncertainty-based depth sampling strategy (PUDS), allowing for adaptive and dynamic adjustments of the depth sampling range. In addition, we employ gated recurrent unit (GRU)-based optimization in the depth refinement step and integrate the extra edge features into the construction of the edge-aware iterative cost volume, ensuring a better reconstruction quality. Below, we provide a detailed explanation of our proposed method.

### 3.1. Preliminaries

Before introducing our method, let us review the general workflow of learning-based multi-view stereo methods. The input image set ${\left\{I_{i}\right\}}_{i=0,1\ldots N-1}$ initially undergoes feature extraction to obtain the corresponding feature maps ${\left\{F_{i}\right\}}_{i=0,1\ldots N-1}$ through a feature extraction network. Then, the differential homography transformation is applied to warp extracted feature maps ${\left\{F_{i}\right\}}_{i=0,1\ldots N-1}$ from the viewpoint of each source image $I_{i}\left(i=1,2\ldots N-1\right)$ to the perspective of a reference image $I_{0}$ pixel-wise and to subsequently construct a cost volume to quantify the correlation and similarity between the source image set $\{I_{i}{\}}_{i=1,2\ldots N-1}$ and the reference image $I_{0}$. In the first depth estimation, we configure the sampling depth hypothesis ${\left\{d_{j}\right\}}_{j=1,2\ldots M}$ at equal intervals within the depth range of the reference image, where *M* represents the total number of hypothetical depth planes. Let the pixel of the reference image be $p_{0}$ and $d_{j}$ be the *j*th hypothetical depth in the sampling depth hypothesis. Then, each pixel coordinate $p_{i}$ in *i*th source image can be calculated as follows:

(1) $$\begin{array}{c} p_{i}=K_{i}R_{i}\left(R_{0}^{T}K_{0}^{-1}p_{0}d_{j}+\left(R_{i}^{T}T_{i}-R_{0}^{T}T_{0}\right)\right), \end{array}$$

where $K_{i}$, $R_{i}$, and $T_{i}$ mean, respectively, the intrinsic matrix, the rotation matrix, and the translation vector under the world coordinates of the *i*th source image. Similarly, $K_{0}$, $R_{0}$, and $T_{0}$ denote the intrinsic matrix, the rotation matrix, and the translation vector under the world coordinates of the reference image. After warping the feature maps to the reference perspective, the correlation of the feature map can measure the probability and the reliability of the $d_{j}$ depth hypothesis. For each reference image $I_{0}$, we construct a feature volume ${\left\{V_{i}\right\}}_{i=1,2\ldots N}$. In the depth hypothesis $d_{j}$, the cost volume $C_{d_{j}}$ can be calculated by the variance of the *N* views as follows:

(2) $$\begin{array}{c} C_{d_{j}}=\frac{\sum_{n=1}^{N}{\left(V_{n}-\bar{V_{n}}\right)}^{2}}{N}, \end{array}$$

where $\bar{V_{n}}$ is the average volume:

(3) $$\begin{array}{c} \bar{V_{n}}=\frac{\sum_{n=1}^{N}V_{n}}{N}, \end{array}$$

Because the raw cost volume usually contains a lot of noise, 3D-UNet is applied for cost volume regularization. In contrast to existing learning-based MVS methods [4,6,8,12], we simplify the four-scale architecture to the light three-scale structure, which helps us to reduce the computation amount and GPU memory consumption. Next, the softmax operation is applied along the depth dimension to normalize the probability volume. Finally, the depth estimation $D_{0}$ can be calculated by multiplying each probability map $p_{d_{j}}$ by the corresponding hypothetical depth value $d_{j}$ as follows:

(4) $$\begin{array}{c} D=\sum_{j=1}^{M}d_{j}\times p_{d_{j}}. \end{array}$$

### 3.2. Light Coarse-Feature-Enhanced Feature Pyramid Network

In recent years, the majority of learning-based multi-view stereo methods [6,24,37,39] have employed the feature pyramid network (FPN) [13] for image feature extraction. This FPN structure effectively captures multi-scale features, making it suitable for a cascaded network architecture [6]. However, the classical FPN module still faces challenges such as a high GPU memory consumption and insufficient feature extraction. To address these issues, we propose a light coarse-feature-enhanced feature pyramid network (LCFE-FPN) for feature extraction. This novel design not only achieves a more lightweight network but also ensures the preservation of the final reconstruction quality.

As shown in Figure 1, the backbone of our LCFE-FPN is primarily based on the classical FPN architecture [13]. The input image is composed of a reference image, $I_{0}$, and N−1 images ${\left\{I_{i}\right\}}_{i=1,2\ldots N-1}$ with the size of W×H. We obtain feature maps at *K* different resolution stages through multi-scale feature extraction. These extracted multi-scale feature maps are represented as $\left\{F_{i}^{k}\in R^{\frac{C}{2^{k-1}}\times \frac{H}{2^{\left(L-k\right)}}\times \frac{W}{2^{\left(L-k\right)}}}|i=1,2\ldots N,k=1,2\ldots L\right\}$, where *L* denotes the total number of resolution stages and *C* represents the number of feature channels. In our experiments, following a similar approach to [6], we set the total number of resolution stages *L* to 3. Specifically, the batch normalization layers in the classic FPN [13] are replaced with in-place-activated batch normalization layers (Inplace-ABN) [14]. Using Inplace-ABN layers [14], which cleverly drop or recompute intermediate buffers as needed, results in a 29% reduction in GPU memory consumption, making our feature extraction module lightweight.

**Figure 2:**
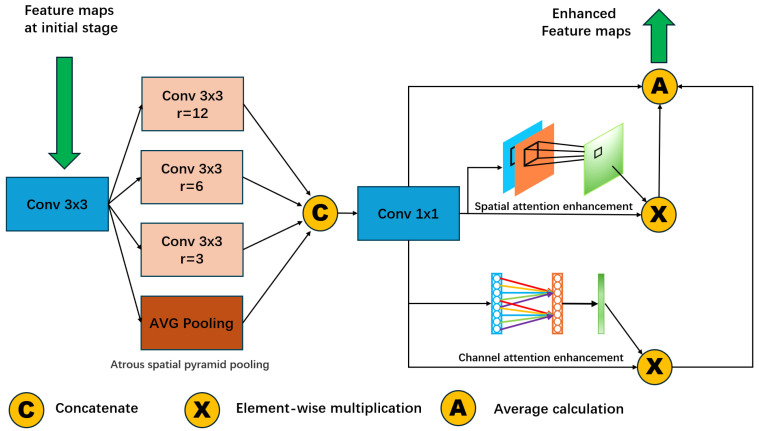
The architecture of the proposed coarse-feature-enhanced (CFE) module. For these feature maps at the coarse stage, we employ dilated convolutions with three different dilation rates (3, 6, and 9) for atrous spatial pyramid pooling. Subsequently, we further enhance these feature maps through the combination of spatial attention and channel attention and, finally, calculate the average as the final enhanced feature map.

To enhance feature extraction at the coarse stage and to improve the model performance, we introduce a novel coarse-feature-enhanced (CFE) module designed to fortify the feature maps in the first stage with the lowest resolution. As depicted in Figure 2, the CFE module employs light atrous spatial pyramid pooling [40] to achieve a larger receptive field for better contextual feature extraction. More specifically, modified lightweight atrous spatial pyramid pooling consists of the following steps: the input feature map passes through a 2D-CNN layer, and then undergoes parallel operations involving three 2D-CNN layers with three different dilation rates (3,6,9) and one average pooling layer. Subsequently, the results are concatenated along the channel dimension and processed through a 1x1 CNN. Later, our coarse-feature-enhanced module (CFE) fuses the enhanced features via channel attention [41] and spatial attention [42] at the lowest-resolution stage. The addition of our CFE module aims to bolster contextual features within the coarse stage and ensures well-initialized depth estimation in the cascade architecture.

### 3.3. Patch-Uncertainty-Based Depth Sampling Strategy

As articulated in related work, uncertainty-based depth sampling strategies have been employed to adjust the sampling distance dynamically during depth refinement. However, learning-based MVS methods with a cascade architecture generally adopt an approach where each stage corresponds to a fixed sampling range. This process overlooks the differences between individual pixels, thereby affecting the final reconstruction quality. At the same time, methods [11,24,43] utilizing uncertainty-based sampling strategies also neglect the influence of its neighborhood on the sampling range for a given pixel, thus disregarding nearby semantic information. We propose a patch-uncertainty-based depth sampling strategy (PUDS) to address these issues above.

**Figure 3:**
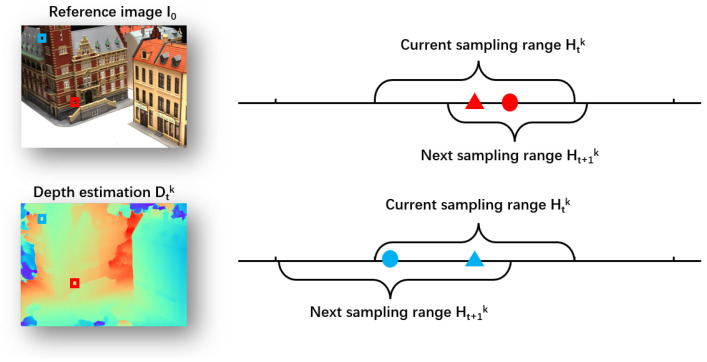
Visualization of the sampling range setting. In this figure, we take two pixels $p_{1}$, $p_{2}$ and their patches as the example. The circle is the current estimation $D_{t}^{k}$, and the triangle is the last estimation $D_{t-1}^{k}$. After the *t*-th optimization, red exhibits the pixel $p_{1}$ with a small patch uncertainty $S_{t}^{k}\left(p_{1}\right)$, thereby setting a smaller sampling range $H_{t+1}^{k}\left(p_{1}\right)$ for the next GRU optimization. In contrast, blue represents the pixel $p_{2}$ with significant patch uncertainty $S_{t}^{k}\left(p_{2}\right)$, necessitating a larger sampling range $H_{t}^{k}\left(p_{2}\right)$ in this case.

As shown in Figure 3, during each GRU optimization, we utilize the PUDS to adaptively adjust the sampling inverse depth hypothesis range. More specifically, for the *t*-th optimization at *k*-th scale stage, we calculate the variation map of the inverse depth $u_{t}^{k}$ using

(5) $$\begin{array}{c} u_{t}^{k}=\frac{\Delta D_{t}^{k}}{D_{t-1}^{k}}, \end{array}$$

followed by min-max pixel-wise normalization:

(6) $$\begin{array}{c} {\hat{u}}_{t}^{k}\left(p\right)=\frac{u_{t}^{k}\left(p\right)-u_{min}}{u_{max}-u_{min}}. \end{array}$$

**Figure 4:**
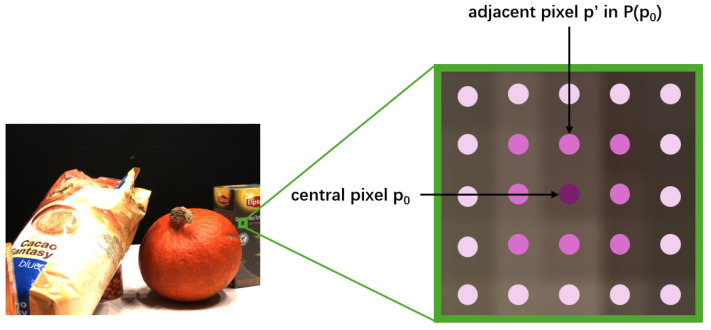
Visualization of patch selection. In this figure, we take a 5 × 5 size patch as an example. In the neighborhood $P(p_{0})$ of the central pixel $p_{0}$, the small dots indicate the magnitude of weights $\mu_{p^{\prime }}$, with darker colors indicating higher weights and lighter colors indicating lower weights.

Subsequently, considering the impact of the neighborhood of each pixel *p* in the normalized variation map, we calculate the coefficient patch uncertainty *S*, serving as a scaling factor to adjust the inverse depth sampling range. As shown in Figure 4, for each pixel *p*, the *k*-th stage patch uncertainty S(p) in the *t*-th optimization is defined by:

(7) $$\begin{array}{c} S_{t}^{k}\left(p\right)=\sum_{p^{\prime }\in P\left(p\right)}\mu_{p^{\prime }}{\hat{u}}_{t}^{k}\left(p^{\prime }\right), \end{array}$$

where *P* represents the neighboring patch of the pixel *p* and $\mu_{p^{\prime }}$ denotes the corresponding weight of the pixel $p^{\prime }$ within the neighboring patch P(p). For each pixel $p^{\prime }$ in the neighboring patch *P*, the weight $\mu_{p^{\prime }}$ attenuates as the distance $∥pp^{\prime }∥$ increases, and the sum of all weights $\mu_{p^{\prime }}$ within the neighbored patch is equal to 1 $\sum_{p^{\prime }\in P\left(p\right)}\mu_{p^{\prime }}=1$. After that, for each pixel *p*, we define the *k*-th stage inverse depth sampling range $H_{t}^{k}$ at the *t*-th optimization as follows:

(8) $$\begin{array}{c} H_{t}^{k}\left(p\right)=\left[\frac{1}{D_{t-1}^{k}\left(p\right)}-S_{t}^{k}\left(p\right)\delta_{k},\frac{1}{D_{t-1}^{k}\left(p\right)}+S_{t}^{k}\left(p\right)\delta_{k}\right], \end{array}$$

where $\delta_{k}$ is a pre-configured sampling distance based on the current stage *k*. The definition of $\delta_{k}$ is as follows:

(9) $$\begin{array}{c} \delta_{k}=\frac{2^{k-1}\left(\frac{1}{d_{min}}-\frac{1}{d_{max}}\right)}{G}, \end{array}$$

where $d_{min}$, $d_{max}$, and *G* are all constant numbers based on the corresponding datasets; we will introduce these parameters more specifically in the following experimental section.

### 3.4. Edge-Aware Iterative Cost Volume

**Figure 5:**
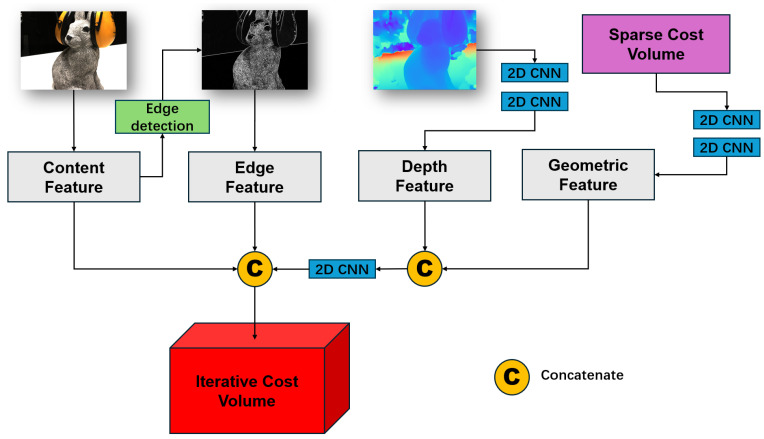
Construction of our edge-aware iterative cost volume. We utilize 2D-CNN layers to extract geometric features from the sparse cost volume and depth features from the previous depth estimation. Additionally, we capture edge features using an edge detection operator. Subsequently, we fuse these four types of features to construct the edge-aware iterative cost volume.

Inspired by [10,16,22,24], we construct an edge-aware iterative cost volume and then utilize GRU-based optimization to refine the depth map. As illustrated in Figure 5, our edge-aware iterative cost volume mainly involves four different types of features: content features, edge features, geometric features, and depth features. From the preceding steps, it can be inferred that we have obtained the cost volume and the depth estimation after multi-scale feature extraction and differential warping. Then, we apply two convolution layers on the cost volume and the depth map, respectively, to extract the content and depth features. Subsequently, we concatenate the content and depth features in the channel dimensions. We then merge them with the geometric features derived from the reference image. A lightweight, classic edge detection operator is employed to extract the edge features from the content features, which are further concatenated along the channel dimensions. This process collectively forms our proposed iterative cost volume.

Next, as shown in Figure 1, we utilize a GRU-based optimizer to process the iterative cost volume and to refine the depth map. Similar to [10], we build a multi-stage architecture to use the multi-scale information further. The GRU optimizer refines the depth map *T* times for each stage *k*, and the output of each GRU optimization is $\Delta D_{t}^{k}$, where *t* denotes the tth GRU optimization. Let the *k*th stage input depth map be $D_{t-1}^{k}$. The refined depth after one optimization can be calculated as follows:

(10) $$\begin{array}{c} D_{t}^{k}=D_{t-1}^{k}+\Delta D_{t}^{k}. \end{array}$$

Suppose the optimization amount *t* equals the pre-configured optimization limit *T*. In that case, the output-refined depth map will be upsampled to next stage k+1 with the size changing from $\frac{H}{2^{\left(L-k\right)}}\times \frac{W}{2^{\left(L-k\right)}}$ to $\frac{H}{2^{\left(L-k-1\right)}}\times \frac{W}{2^{\left(L-k-1\right)}}$, serving as the initial input depth map for stage k+1.

### 3.5. Loss Function

We consider both the loss of initial depth estimation and the loss of refined depth estimation at each stage after each optimization. L1-normal represents the distance between each depth estimation and the corresponding ground truth depth map during training. Similar to other learning-based multi-view stereo methods [4,5,6], we consider pixels with a valid label in the ground truth depth map to avoid the potential negative impact from the fact that not every pixel in the ground truth depth map is valid. Thus, each loss $L_{t}^{k}$ can be calculated as follows:

(11) $$\begin{array}{c} L_{t}^{k}=\sum_{p\in P_{valid}}{∥D^{k}\left(p\right)-D_{t}^{k}\left(p\right)∥}_{1}, \end{array}$$

where $D^{k}\left(p\right)$ represents the pixel in ground truth depth map at the *k*-th resolution stage. Subsequently, the final loss $Loss_{final}$ can be calculated as follows:

(12) $$\begin{array}{c} L_{final}=L_{0}+\sum_{k=1}^{3}\sum_{t=1}^{T}\lambda^{t-T}L_{t}^{k}. \end{array}$$

Here, $L_{0}$ represents the loss of our first estimated depth map without GRU optimization, and $\lambda^{t-T}$ is a coefficient that exponentially decays with each optimization. We set the constant λ to 0.9 in our experiments.

## 4. Experiments

We have evaluated our approach on established multi-view stereo benchmark datasets, namely the DTU dataset [17] and the Tanks and Temples dataset [18]. A series of experiments were conducted to verify the efficacy of our approach proposed in this paper.

### 4.1. Datasets

#### 4.1.1. DTU

The DTU dataset [17] is an indoor dataset aimed at multi-view stereo evaluation. This dataset consists of 124 scenes captured by a structured light scanner mounted on an industrial robot arm under various lighting conditions. In the DTU dataset, each scene contains 49 views from distinct camera poses along the same camera motion, and each view contains an RGB photo and the corresponding ground truth depth map. The DTU dataset also provides ground truth 3D point clouds for evaluation.

#### 4.1.2. Tanks and Temples

The Tanks and Temples dataset [18] is an outside-lab dataset including outdoor and indoor scenes. Each scene is a video sequence captured under realistic conditions by a moving industrial laser scanner. The Tanks and Temples dataset is divided into intermediate and advanced subsets. The intermediate subset features sculptures, large vehicles, and house-scale buildings with exterior-looking camera trajectories. The advanced subset includes expansive indoor and outdoor scenes with complex geometries and diverse camera paths.

#### 4.1.3. BlendedMVS

The BlendedMVS dataset [44] is a recently released large-scale dataset for learning-based multi-view stereo which contains over 17 k high-resolution images in various scenes.

### 4.2. Evaluation Metrics

We utilized metrics based on accuracy and completeness to evaluate the quality of the final reconstruction results. Accuracy represents the distance between each estimated point cloud and the corresponding ground truth point cloud, while completeness is measured from the ground truth point cloud to the estimated point cloud. Meanwhile, we also calculate the F1 score, considering both completeness and accuracy, as the evaluation metric in the experiments on the Tanks and Temples dataset [18].

### 4.3. Implementation Details

Following general practice like other learning-based multi-view stereo methods [5,6,8,10,12], we trained our model on the DTU [17] training set and evaluated it on the evaluation set with the same parameter configuration. We also fine-tuned our model on the BlendedMVS [44] dataset and evaluated the results on the Tanks and Temples [18] dataset. In the calculation of pixel weights $\mu_{p}$ within the patch $P(p_{0})$ for the PUDS, we used the Chebyshev distance as metric of the distance $∥pp_{0}∥$ from pixel *p* to the center pixel $p_{0}$ in the patch $P(p_{0})$. In the experiment on patch size, for the 3 × 3, 5 × 5, and 7 × 7 patch sizes, the pixel weight $\mu_{p}$ in each patch $P(p_{0})$ was set in ascending order according to the distance $∥pp_{0}∥$. It was, respectively, set to ($\frac{1}{2}$, $\frac{1}{16}$), ($\frac{1}{3}$, $\frac{1}{24}$, $\frac{1}{48}$) and ($\frac{1}{4}$, $\frac{1}{32}$, $\frac{1}{64}$, $\frac{1}{96}$). In all experiments conducted in this paper, the intrinsic and extrinsic camera parameters used were obtained from the datasets [17,18,44] directly.

#### 4.3.1. Training

The resolution of the input images was 640 × 512, and the number of input images was *N* = 5. We set 48 evenly spaced planes for depth hypotheses in the first depth estimation. Subsequently, we set the number of depth hypotheses to 4 for iterative cost volume construction during depth refinement. In the first depth refinement in each stage, we defined the minimum sampling distance $\delta_{0}$ for the inverse depth as:

(13) $$\begin{array}{c} \delta_{0}=\frac{\left(1/d_{\min}-1/d_{\max}\right)}{G}, \end{array}$$

where $d_{\min}$ = 425 mm, $d_{\max}$ = 935 mm, and *G* = 384 for the DTU dataset [17]. We configured the optimization number *T* for GRU-based optimization as 3 for each stage. We implemented our method in PyTorch [45] and trained the model using the AdamW optimizer with a OneCycleLR schedule and a maximum learning rate of 0.001. We trained our model on a single NVIDIA GeForce RTX 3090 GPU sourced from NVIDIA Corporation in Santa Clara, California, United States, with a batch size 8.

#### 4.3.2. Evaluation

For the evaluation on the DTU dataset [17], we utilized the provided MATLAB evaluation program and the ground truth point cloud. The resolution of input images was 1600 × 1184, and the number of input images was *N* = 5. For the evaluation on the Tanks and Temples dataset [18], we uploaded the final reconstruction result to the Tanks and Temple [18] official website and published the online evaluation results on the leaderboard. The resolution of input images was 1920 × 1056, and the number of input images was *N* = 7. Like other learning-based multi-view stereo methods, we leveraged the photometric and geometric consistency to generate filters for the depth map output and then fused these to obtain the final point cloud results.

#### 4.3.3. Fine-Tuning

Before the evaluation on the Tanks and Temples dataset [18], we trained the model on the BlendedMVS dataset [44] for 16 epochs. During fine-tuning, the resolution of input images was set to 768 × 576, and the number of input images was set to *N* = 5. Unlike training on the DTU dataset [17], we assigned 96 evenly spaced planes for depth hypotheses in the first depth estimation. Meanwhile, we set *G* = 768 for fine-tuning.

### 4.4. Results

We conducted a comparative analysis of our method with other existing multi-view stereo methods [1,4,5,6,8,9,10,11,19,20,21,22,23,24], focusing on criteria such as the reconstruction quality, GPU memory consumption, and the running time. For the sake of ensuring a fair comparison, we used the same experimental configurations across all methods. We uniformly employed original-resolution images and set the input view number to 5 for the DTU dataset [17] evaluation and 7 for the Tanks and Temples dataset [18] evaluation.

#### 4.4.1. Performance on the DTU Benchmark

**Figure 6:**
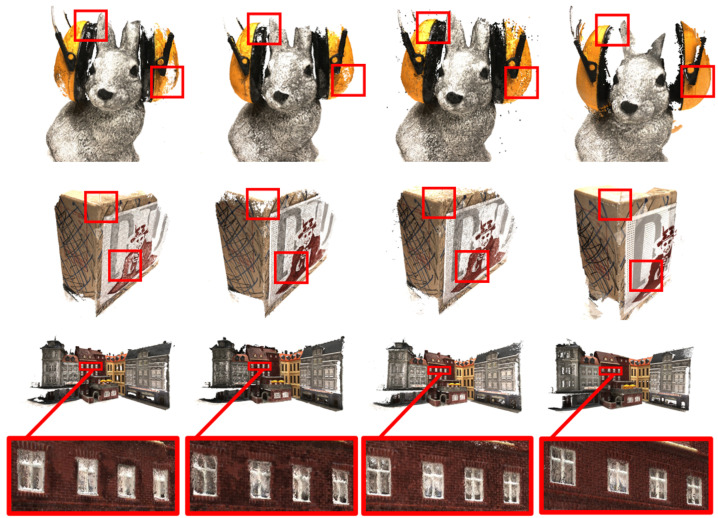
The point cloud quality comparison. From top to bottom, each row represents scan33, scan13, and scan23 in the DTU dataset [17]. From left to right, the point cloud results correspond to PatchmatchNet [5], CasMVSNet [6], our method, and the ground truth.

For a comparison using the DTU dataset [17], we compared our results with some traditional methods [1,19,20,21] and learning-based methods [4,5,6,8,9,10,11,22,23,24]. For a quantitative evaluation, we use official MATLAB codes to calculate the accuracy and completeness. As the quantitative results show in Table 1, our method not only excels in efficiency, as demonstrated by the lower GPU memory consumption and faster running time, but also delivers a competitive performance in terms of the reconstruction quality. In addition, we present quality comparisons using the DTU dataset [17] in Figure 6. Compared to PatchmatchNet [5] and CasMVSNet [6], it is evident that the point clouds generated by our method exhibit a superior performance in capturing finer details.

#### 4.4.2. Performance on Tanks and Temples Benchmark

**Figure 7:**
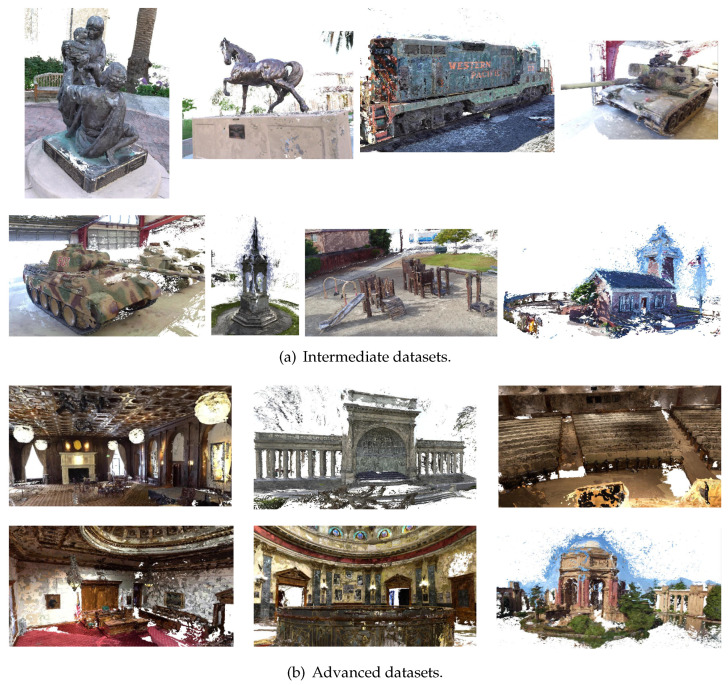
Reconstruction results on the Tanks and Temples dataset.

**Figure 8:**
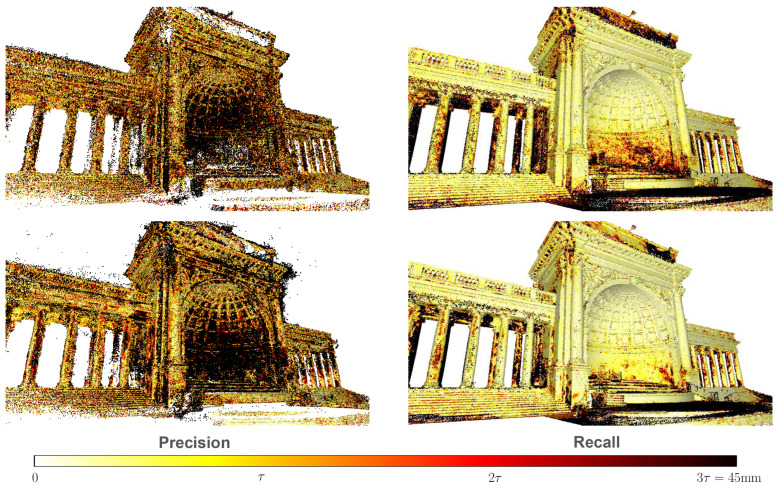
Error visualization comparison on the temple scene of the Tanks and Temples dataset. The upper row shows the results of Transmvsnet [37], while the lower row displays our results.

For a comparison using Tanks and Temples dataset, we compared our results with the widely used open-source reconstruction software COLMAP [1] and well-known established learning-based MVS methods [4,5,6,8,9,10,11,22,23,24,37]. For quantitative evaluations, we submitted our reconstruction results to the Tanks and Temples [18] website, publishing the mean F-score on its leaderboard. As indicated in Table 2, in the qualitative evaluation, our method achieved a commendable overall performance. Notably, it ranks first on the advanced subset compared to other methods, demonstrating our model’s robustness and generalization capabilities. Figure 7 presents qualitative visualizations showcasing the robust reconstructive capabilities of our algorithm, particularly emphasizing its effectiveness in handling large-scale outdoor scenes. In addition, we further compare our method with the state-of-the-art TransMVSNet [37] method. Taking the temple scene from the Tanks and Temples advanced dataset as the example, as depicted in Figure 8, it can be observed that our method exhibits a superior performance in reconstruction completeness.

### 4.5. Ablation Study

#### 4.5.1. Effect of Each Component

Table 3 presents the ablation results of our proposed method. The evaluation results of the baseline method [10] are reproduced with the same parameter configuration as the others. The LCFE-FPN model enhances features at the coarse stage, resulting in a 3.6% improvement in accuracy. Notably, the lightweight structure of LCFE-FPN significantly reduces GPU memory consumption from 3.1 GB to 2.2 GB, approximately 29%. Meanwhile, using the proposed patch-uncertainty-based sampling (PUDS) strategy enables the adaptive setting of a more accurate sampling range, leading to a 7.4% improvement in completeness. Additionally, incorporating additional edge features (EFs) in iterative cost volume construction leads to more attention to detail, thereby improving the model’s performance in terms of completeness and overall performance.

#### 4.5.2. Ablation in Feature Extraction

In this section, we conduct ablation experiments on the feature extraction step of our method in this study, comparing our light, coarse-feature-enhanced feature pyramid network (LCFE-FPN) with the traditional FPN. During this experiment, we only modified the feature extraction step without altering the other steps of the algorithm. As shown in Table 4, utilizing the LCFE-FPN in the feature extraction step enhances the reconstruction quality of the model while reducing the memory consumption. Moreover, it should be noted that the resulting increase in algorithm runtime is also slight and acceptable.

#### 4.5.3. Ablation in Sampling Strategy

This section compares the proposed patch-uncertainty-based depth sampling strategy (PUDS) with the commonly used uniform sampling strategy (US). As demonstrated in Table 5, the utilization of the PUDS significantly enhances the quality of the final point cloud during the GRU optimization process. Furthermore, in terms of GPU consumption and algorithm runtime, the PUDS also exhibits a comparable performance.

#### 4.5.4. Ablation in Iterative Cost Volume Construction

During iterative cost volume construction in the depth refinement step, we also tested the effect of edge features (EFs). Table 6 presents the results. Note that the utilization of EFs can notably enhance the overall performance regarding the reconstruction quality, particularly in completeness. At the same time, it is worth noting that incorporating edge features will also lead to a slight increase in algorithmic runtime and memory consumption.

#### 4.5.5. Edge Extraction Operator Selection

In this section, we conduct additional experiments to evaluate the individual impact of each component in our approach. Moreover, we explore the effects of patch size in depth sampling and the selection of operators in edge feature extraction. All ablation experiments were conducted using the DTU dataset [17]. In this experiment, we selected four well-known operators [15,46,47,48] in edge detection as alternatives. Keeping the same experimental configuration, we evaluated the final reconstruction results on the DTU dataset [17]. As depicted in Table 7, the Sobel [15] operator emerges as the ultimate winner, closely followed by the Prewitt [46] operator in second position.

#### 4.5.6. Size of the Patch

Furthermore, to evaluate the impact of the patch size in PUDS, we tested four different sizes: 1 × 1, 3 × 3, 5 × 5, and 7 × 7. The 1 × 1 patch size implies that PUDS does not consider uncertainty at the patch level but focuses on each pixel individually. As shown in Table 8, a patch size 3 × 3 demonstrates improved evaluation performance in the DTU dataset [17].

## 5. Conclusions

In this paper, we introduce a novel learning-based multi-view stereo method, presenting significant contributions to address the key challenges in the field. Our main accomplishments can be summarized as follows: Firstly, we propose a novel, light, coarse-feature-enhanced feature pyramid network designed to effectively balance the GPU memory consumption and the final reconstruction quality. Secondly, in the depth refinement phase, we put forward a novel patch-uncertainty-based sampling strategy that can calculate the patch-wise uncertainty based on each pixel’s depth variation and adaptively adjust the inverse depth sampling range. This strategy ensures adaptive and dynamic assignment of different sampling ranges for each pixel. In addition, we integrate edge information extracted by the Sobel operator into iterative cost volume construction, further enhancing our model’s performance. Furthermore, through comparative evaluations against other learning-based multi-view stereo methods, our approach demonstrates a competitive performance, excelling not only in terms of GPU consumption but also in terms of producing high-quality reconstructions of benchmark datasets such as the DTU dataset and the Tanks and Temples dataset.

The method proposed in this paper also has some limitations and deficiencies. In the feature extraction step, compared to transformer-based methods, our LCFE-FPN benefits from a relatively simple structure, which gives it advantages in terms of memory usage and runtime speeds. However, our final reconstruction quality is lower overall than that of transformer-based methods. In the future, we can explore using a lightweight transformer for feature extraction to achieve better results. On the other hand, in outdoor scenes, learning-based MVS methods struggle to estimate the depth of the sky and its boundaries, which affects the final reconstruction quality. If we remove the sky from images before feature extraction, this might enhance the model’s performance in outdoor scenarios and the generalization capability.

In the future, we aim to integrate the proposed method with the SLAM system, further refining its efficiency. This integration will facilitate its deployment within automated production environments, enabling real-time dense reconstruction of three-dimensional scenes close to visual robotic arms. This advancement will enhance automatic positioning, obstacle avoidance, and gripping operation capabilities.

## Figures and Tables

**Table 1:** Quantitative comparison on the DTU dataset (the lower, the better). The **bold** indicates the best, while the underlined indicates the second best.

Method	Acc. (mm)	Comp. (mm)	Overall (mm)	Mem. (GB)	Time (s)
COLMAP [1]	0.400	0.664	0.532	-	-
Tola [20]	0.342	1.190	0.766	-	-
Furu [19]	0.613	0.941	0.777	-	-
Gipuma [21]	**0.283**	0.873	0.578	-	-
MVSNet [4]	0.396	0.527	0.462	-	-
R-MVSNet [22]	0.385	0.459	0.417	-	-
Fast-MVSNet [23]	0.336	0.403	0.370	7.0	0.52
Vis-MVSNet [9]	0.369	0.361	0.365	5.6	0.61
CVP-MVSNet [8]	0.296	0.406	0.351	8.8	1.51
CasMVSNet [6]	0.346	0.351	0.348	5.3	0.55
UCS-Net [11]	0.338	0.349	0.344	6.6	0.54
PatchmatchNet [5]	0.427	**0.277**	0.352	3.6	0.29
Effi-MVSNet [10]	0.321	0.313	0.317	3.1	**0.19**
IterMVS [24]	0.373	0.354	0.363	4.7	0.20
TransMVSNet [37]	0.321	0.289	**0.305**	3.0	0.71
Ours	0.320	0.307	0.314	**2.2**	0.27

**Table 2:** Quantitative comparison on the Tanks and Temples dataset (the higher, the better). The **bold** indicates the best, while the underlined indicates the second best.

Method	Intermediate									Advanced						
	Mean	Fam.	Fran.	Hor.	L.H.	M60	Pan.	P.G.	Tra.	Mean	Aud.	Ball.	Cou.	Mus.	Pal.	Tem.
COLMAP [1]	42.14	50.41	22.25	25.63	56.43	44.83	46.97	48.53	42.04	27.24	16.02	25.23	34.70	41.51	18.05	27.94
MVSNet [4]	43.48	55.99	28.55	25.07	50.79	53.96	50.86	47.90	34.69	-	-	-	-	-	-	-
Fast-MVS [23]	47.39	65.18	39.59	34.98	47.81	49.16	46.20	53.27	42.91	-	-	-	-	-	-	-
R-MVSNet [22]	48.40	69.96	46.65	32.59	42.95	51.88	48.80	52.00	42.38	24.91	12.55	29.09	25.06	38.68	19.14	24.96
Vis-MVSNet [9]	60.03	77.40	60.2	47.07	**63.44**	62.21	57.28	**60.54**	52.07	33.78	20.79	38.77	32.45	44.20	28.73	37.70
CVP-MVSNet [8]	54.03	76.50	47.74	36.34	55.12	57.28	54.28	57.43	47.54	-	-	-	-	-	-	-
CasMVSNet [6]	56.84	76.37	58.45	46.26	55.81	56.11	54.06	58.18	49.51	31.12	19.81	38.46	29.10	43.87	27.36	28.11
UCS-Net [11]	54.83	76.09	53.16	43.03	54.00	55.60	51.49	57.38	47.89	-	-	-	-	-	-	-
PatchmatchNet [5]	53.15	66.99	52.64	43.24	54.87	52.87	49.54	54.21	50.81	32.31	23.69	37.73	30.04	41.80	28.31	32.29
Effi-MVSNet [10]	56.88	72.21	51.02	51.78	58.63	58.71	56.21	57.07	49.38	34.39	20.22	42.39	33.73	45.08	29.81	35.09
IterMVS [24]	56.22	73.57	54.39	50.16	54.70	58.50	52.54	54.51	51.38	33.24	22.95	38.74	30.64	43.44	28.39	35.27
TransMVSNet [37]	**63.52**	**80.92**	**65.83**	**56.94**	62.54	**63.06**	**60.00**	60.20	**58.67**	37.00	24.84	**44.59**	34.77	46.49	**34.69**	36.62
Ours	60.01	77.97	62.26	52.77	60.24	58.32	55.80	58.36	54.38	**37.67**	**28.70**	42.65	**35.47**	**47.80**	31.29	**40.12**

**Table 3:** Ablation evaluation results on the DTU dataset. The **bold** indicates the best.

Methods	Acc. (mm)	Comp. (mm)	Overall. (mm)
baseline	0.326	0.324	0.325
+ LCFE	**0.314**	0.329	0.322
+ PUDS	0.337	**0.300**	0.319
+ LCFE + PUDS	0.319	0.315	0.317
+ LCFE + PUDS + EF (Ours)	0.320	0.307	**0.314**

**Table 4:** Effect of the LCFE-FPN on the DTU dataset. The **bold** indicates the best.

Methods	Acc. (mm)	Comp. (mm)	Overall (mm)	Mem. (GB)	Time (s)
FPN	0.345	**0.301**	0.323	3.6	**0.25**
LCFE-FPN	**0.320**	0.307	**0.314**	**2.2**	0.27

**Table 5:** Effect of the PUDS on the DTU dataset. The **bold** indicates the best.

Methods	Acc. (mm)	Comp. (mm)	Overall (mm)	Mem. (GB)	Time (s)
US	0.326	0.324	0.325	**2.2**	**0.26**
PUDS	**0.320**	**0.307**	**0.314**	**2.2**	0.27

**Table 6:** Comparison of the impact of edge features on the DTU dataset. The **bold** indicates the best.

Methods	Acc. (mm)	Comp. (mm)	Overall (mm)	Mem. (GB)	Time (s)
No EF	**0.319**	0.315	0.317	**2.1**	**0.24**
EF	0.320	**0.307**	**0.314**	2.2	0.27

**Table 7:** Different operators in edge feature extraction. The **bold** indicates the best.

Operator	Acc. (mm)	Comp. (mm)	Overall (mm)
Prewitt [46]	0.341	**0.297**	0.319
Scharr [47]	0.333	0.318	0.326
Roberts [48]	0.326	0.412	0.369
Sobel (Ours) [15]	**0.320**	0.307	**0.314**

**Table 8:** Different patch sizes in PUDS. The **bold** indicates the best.

Patch Size	Acc. (mm)	Comp. (mm)	Overall (mm)
1 × 1	0.331	0.348	0.330
3 × 3 (Ours)	0.320	**0.307**	**0.314**
5 × 5	**0.319**	0.314	0.317
7 × 7	0.323	0.419	0.371

## Data Availability

The data are contained within the article.
